# Xanthohumol improves cognition in farnesoid X receptor-deficient mice on a high-fat diet

**DOI:** 10.1242/dmm.049820

**Published:** 2022-11-25

**Authors:** Payel Kundu, Ines L. Paraiso, Jaewoo Choi, Cristobal L. Miranda, Chrissa Kioussi, Claudia S. Maier, Gerd Bobe, Jan F. Stevens, Jacob Raber

**Affiliations:** ^1^ Department of Behavioral Neuroscience, Oregon Health and Science University, Portland, OR 97239, USA; ^2^ Linus Pauling Institute, Oregon State University, Corvallis, OR 97331, USA; ^3^ Department of Pharmaceutical Sciences, Oregon State University, Corvallis, OR 97331, USA; ^4^ Department of Chemistry, Oregon State University, Corvallis, OR 97331, USA; ^5^ Department of Animal and Rangeland Sciences, Oregon State University, Corvallis, OR 97331, USA; ^6^ Departments of Neurology and Radiation Medicine, Division of Neuroscience, Oregon National Primate Research Center, Oregon Health and Science University, Portland, OR 97239, USA

**Keywords:** Western diet, Hippocampus, Water maze, Ceramides, Diacylglycerols, Farnesoid X receptor

## Abstract

Xanthohumol (XN) improves cognition of wild-type rodents on a high-fat diet (HFD). Bile acids and ceramide levels in the liver and hippocampus might be linked to these effects. XN modulates activity of the nuclear farnesoid X receptor (FXR; also known as NR1H4), the primary receptor for bile acids. To determine the role of FXR in the liver and intestine in mediating the effects of XN on cognitive performance, mice with intestine- and liver-specific FXR ablation (FXR^Intestine−/−^ and FXR^Liver−/−^, respectively) on an HFD or an HFD containing XN were cognitively tested. XN improved cognitive performance in a genotype- and sex-dependent manner, with improved task learning in females (specifically wild-type), reversal learning in males (specifically wild-type and FXR^Intestine−/−^ mutant) and spatial learning (both sexes). XN increased hippocampal diacylglycerol and sphingomyelin levels in females but decreased them in males. XN increased the ratio of shorter-chain to longer-chain ceramides and hexaceramides. Higher diacylglycerol and lower longer-chain ceramide and hexaceramide levels were linked to improved cognitive performance. Thus, the beneficial sex-dependent cognitive effects of XN are linked to changes in hippocampal diacylglycerol and ceramide levels.

This article has an associated First Person interview with the first author of the paper.

## Teaser

**Summary:** In mice, the beneficial sex-dependent cognitive effects of xanthohumol under the conditions of a high-fat diet are associated with changes in hippocampal ceramides and diacylglycerols.

## INTRODUCTION

Fat consumption, specifically a diet high in saturated fatty acids, contributes substantially to the obesity epidemic as well as to the development of cognitive impairment and dementia (Freeman et al., 2013; Ruan et al., 2018; Smith et al., 2011). Diet-induced obesity leads to impaired insulin signaling and subsequent dysregulation of glucose utilization. Recent US estimates indicate that 71.6% of American adults are overweight or obese, and that most Americans are eating more than the recommended amount of dietary fat (Hales et al., 2018; https://ourworldindata.org/diet-compositions). A high-fat diet (HFD) can lead to cognitive impairments. Early studies by Greenwood and Winocur demonstrated that young rodents fed a diet with 40% fat content showed impaired spatial and working memory compared to rodents fed a low-fat chow (Greenwood and Winocur, 1990). These effects were even observed in studies involving a 40% polyunsaturated fat diet. The authors later showed that a diet containing as little as 20% saturated fatty acid (SFA) content was enough to impair cognition in young rodents (Greenwood and Winocur, 2005). Since those initial studies, a large body of work, including work from our group, has confirmed the finding that an HFD can impair cognition, in both rodents and humans (Freeman et al., 2013; Johnson et al., 2016; Kalmijn, 2000; Miranda et al., 2018; Smith et al., 2011; Winocur and Greenwood, 2005; Zimmerman et al., 2021).

Xanthohumol (XN) is a prenylated flavonoid found exclusively in the hop plant *Humulus lupulus* (Stevens and Page, 2004). XN improves metabolic outcomes, including fasting glucose and plasma triglyceride levels, in a rodent model of obesity and metabolic syndrome (Legette et al., 2013; Miranda et al., 2018; Nozawa, 2005). The beneficial effects of XN do not necessarily require a significant reduction in body weight gain in response to an HFD (Miranda et al., 2018). XN significantly reduces plasma markers of reactive oxygen species, reduces peripheral markers of dysfunctional lipid oxidation, suppresses the activation of sterol regulatory element-binding proteins and increases uncoupled cellular respiration *in vitro* in obese male rats, all of which could mediate the beneficial effects of XN on metabolic outcomes (Kirkwood et al., 2013; Miyata et al., 2015). Additionally, dietary supplementation with XN has been shown to improve spatial, as well as reversal, learning in a Morris water maze, in young wild-type mice (Miranda et al., 2018; Zamzow et al., 2014), and to reduce cognitive injury associated with alterations in the gut microbiome in a mouse model of Alzheimer's disease transgenically expressing human amyloid precursor protein and presinilin 1 (Liu et al., 2022). Therefore, XN is a promising therapeutic compound for its beneficial effects on metabolism and cognition as well as its excellent safety profile, with no detectable toxicity in mice treated with doses of up to 1000 mg/kg (Dorn et al., 2010). XN also suppresses inflammation in chondrocytes and ameliorates osteoarthritis in mice (Chen et al., 2021).

XN binds to the nuclear farnesoid X receptor (FXR; also known as NR1H4) and activates it *in vivo* (Nozawa, 2005; Yang et al., 2016)*.* XN acts as both an agonist and an indirect modulator of FXR, possibly through upregulation of *Cyp7a1*, the rate-limiting enzyme in the classic bile synthesis pathway (Vlahcevic et al., 1999). FXR is a member of the nuclear hormone receptor superfamily and is the primary endogenous receptor for bile acids (Parks et al., 1999). Bile acids are an important regulator of cholesterol, lipid and glucose homeostasis (Trauner et al., 2010). In addition to their well-known roles in metabolism, they act as active signaling molecules with systemic endocrine functions. Activation of the FXR by bile acids initiates a negative-feedback loop inhibiting the synthesis of bile acids as well as *de novo* synthesis of glycogen and lipids (Trauner et al., 2010). FXR activation plays a role in insulin sensitivity, carbohydrate metabolism and hepatic triglyceride homeostasis (Jiao et al., 2015; Stayrook et al., 2005; Trauner et al., 2010). Thus, FXR is an attractive therapeutic target for improving metabolic outcomes (Zhang et al., 2021). The gene encoding FXR (*Nr1h4*) is highly expressed in the liver and intestine and is evolutionarily conserved, with a high degree of similarity across mammals (Zhu et al., 2011).

In this study, we used mice with intestine- and liver-specific FXR ablation (FXR^Intestine−/−^ and FXR^Liver−/−^, respectively) on an HFD to determine the role of FXR in the liver and intestine in mediating the effects of XN on cognitive performance. Elevated ceramide levels are linked to obesity, insulin resistance and mitochondrial dysfunction (Bikman and Summers, 2011; Summers, 2006; Xie et al., 2017). Peripheral ceramides readily cross the blood–brain barrier and may be a link between diet-induced obesity and cognitive impairment (Zimmerman et al., 2001). FXR in the intestine regulates ceramide synthesis (Jiang et al., 2015; Xie et al., 2017). As in young wild-type male mice, XN treatment decreases ceramide levels in the liver and hippocampus, liver and hippocampal ceramide levels are related to each other, and hippocampal ceramide levels are associated with spatial memory retention in the water maze (Paraiso et al., 2020), we also analyzed ceramide levels in the liver, hippocampus and plasma. Most previous XN HFD studies involved only male rodents; therefore, we included both male and female mice to assess any potential sex-dependent XN effects.

## RESULTS

### XN levels in wild-type, FXR^Liver−/−^ and FXR^Intestine−/−^ mice

Fecal XN levels were comparable in wild-type and FXR^Intestine−/−^ mice, but two XN metabolites [isoxanthohumol (IX) and desmethylxanthohumol (DMX)] were lower in FXR^Intestine−/−^ mice than in wild-type mice (Table 1). As we reported earlier (Paraiso et al., 2021), in FXR^Liver−/−^ and wild-type mice, there were no genotype differences in plasma XN, IX or DXN levels. In FXR^Liver−/−^ and wild-type female and male mice, there were no genotype differences in the levels of XN or 8-prenylnaringenin (8PN) in the liver, but, in females, IX levels in the liver were lower in FXR^Liver−/−^ mice than in wild-type mice (Paraiso et al., 2021).

**Table 1.**
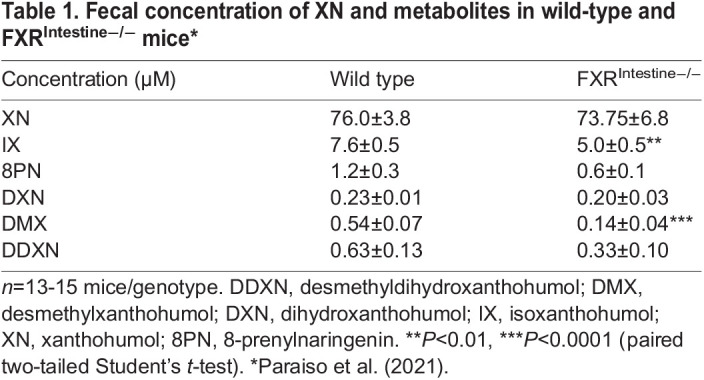
Fecal concentration of XN and metabolites in wild-type and FXR^Intestine−/−^ mice*

### XN supplementation improves task learning of wild-type females during visible platform training

Mice were tested for spatial learning and memory in the water maze. Swim speed was not affected by XN, genotype, sex or their interactions during visible platform trials (Table 2). Therefore, latency to reach the platform and cumulative distance to the platform could be used as performance measures without considering potential treatment effects on swim speeds.

**Table 2.**
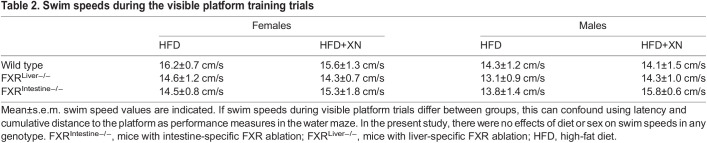
Swim speeds during the visible platform training trials

**Fig. 1.**
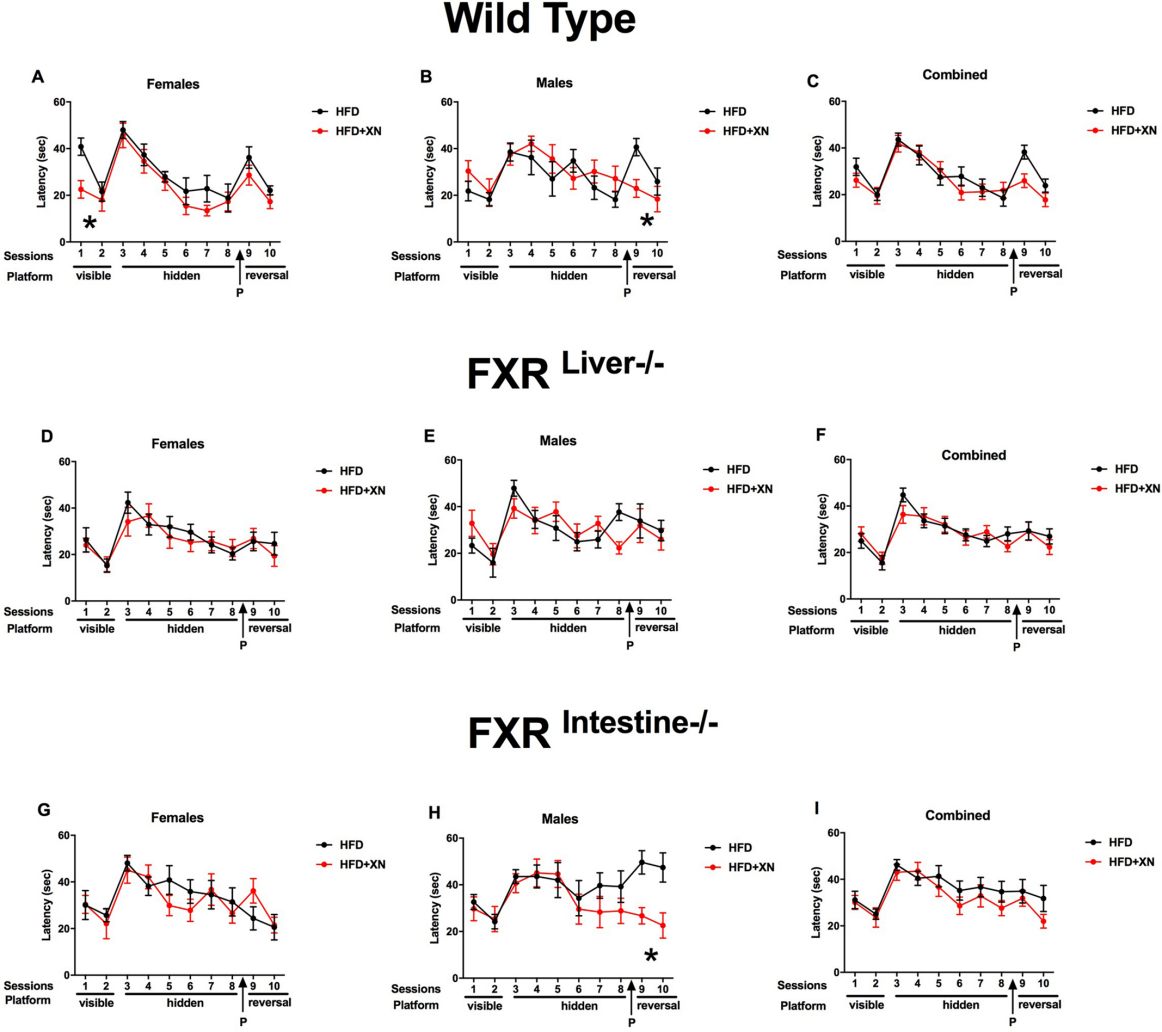
**Latency to find the platform during training sessions.** (A-I) Females are represented in the leftmost graphs (A,D,G) and males in the middle graphs (B,E,H); the sexes are combined in the rightmost graphs (C,F,I). A lower latency indicates better performance. In visible platform trials, xanthohumol (XN) improved performance in wild-type females, but impaired it in males. In reversal trials, XN improved performance in both sexes in wild-type mice. In mice with intestine-specific FXR ablation (FXR^Intestine−/−^ mice), XN impaired performance in females, but dramatically improved reversal learning in males. *P<0.05 for effects of XN on water maze performance (repeated-measures ANOVA sessions 9 and 10). HFD, high-fat diet.

**Fig. 2.**
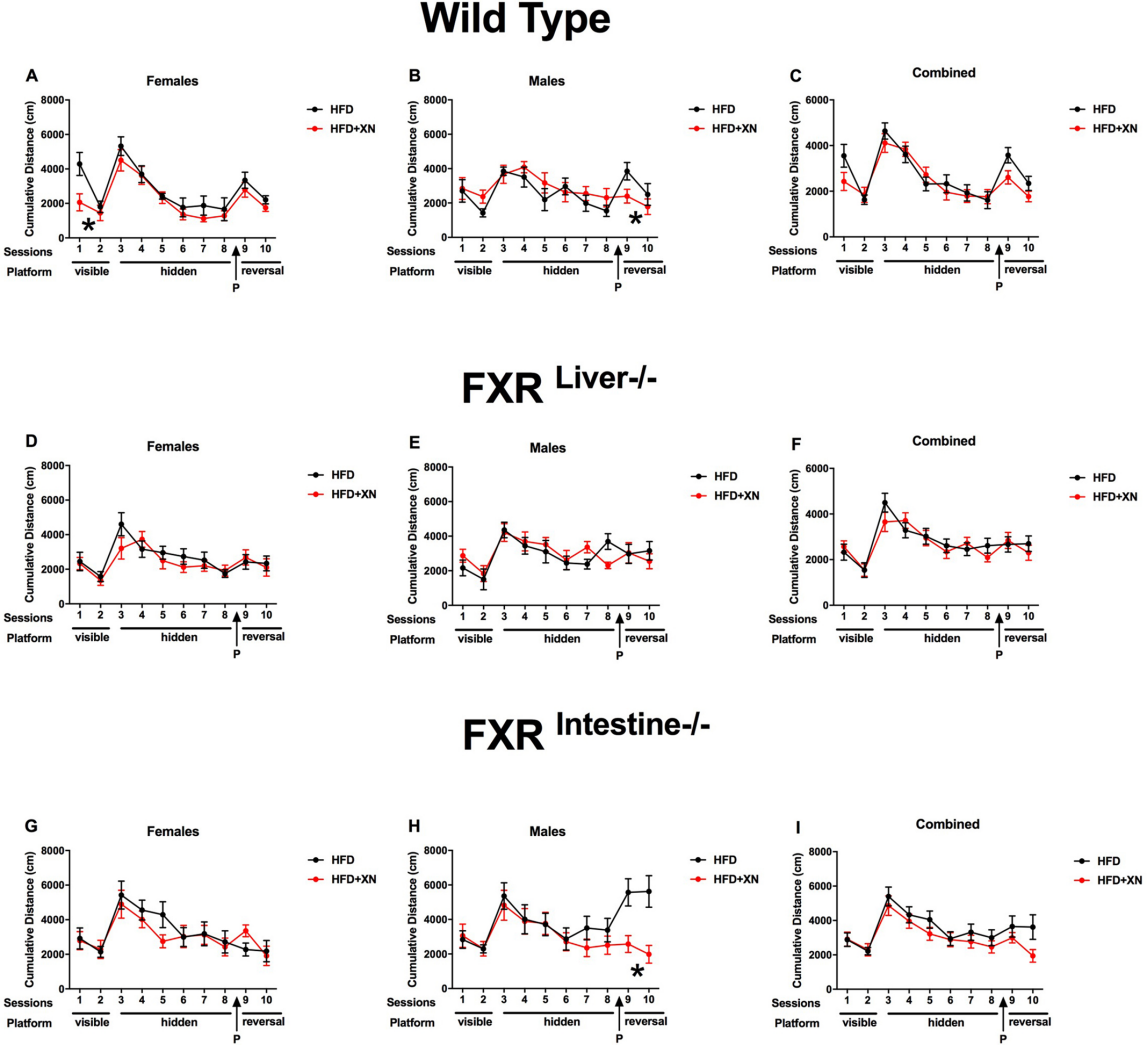
**Cumulative distance to the platform during training sessions.** (A-I) Females are represented in the leftmost graphs (A,D,G) and males in the middle graphs (B,E,H); the sexes are combined in the rightmost graphs (C,F,I). A lower cumulative distance indicates better performance. In visible platform trials, XN improved performance in wild-type females, but impaired it in males. In reversal trials, XN improved performance in both sexes in wild-type mice. In FXR^Intestine−/−^ mice, XN impaired performance in females, but dramatically improved reversal learning in males. *P<0.05 for effects of XN on water maze performance (repeated-measures ANOVA sessions 9 and 10).

**Fig. 3.**
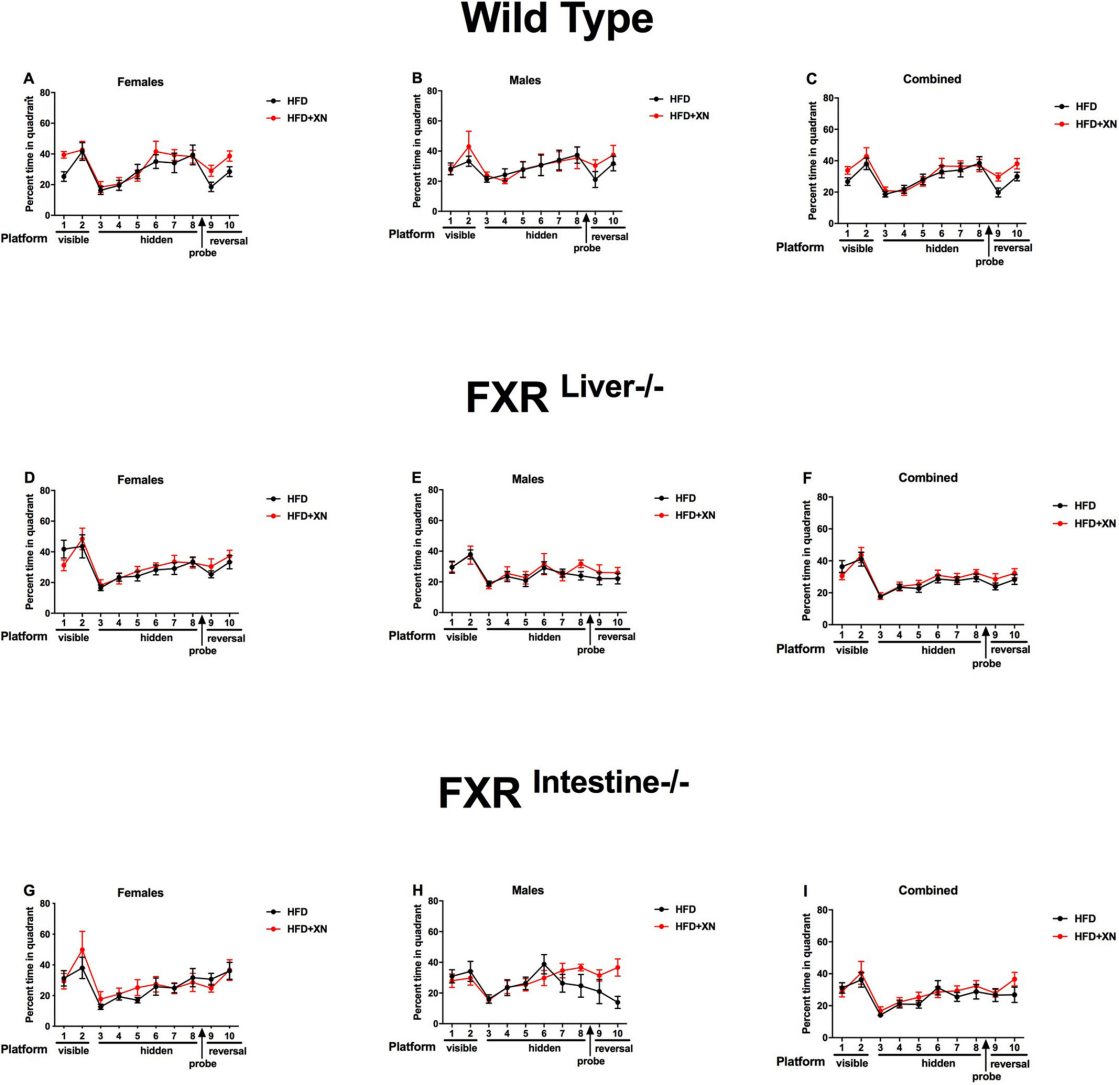
**Percentage of time spent in the target quadrant during training sessions.** (A-I) Females are represented in the leftmost graphs (A,D,G) and males in the middle graphs (B,E,H); the sexes are combined in the rightmost graphs (C,F,I). A higher percentage of time spent in the target quadrant (the quadrant containing the hidden platform) indicates better performance. In reversal trials, XN improved performance in both sexes in wild-type mice. In FXR^Intestine−/−^ mice, XN impaired performance in females, but improved reversal learning in males.

Task learning improved during the two visible platform trials, as mice reached the platform faster earlier and with a lower cumulative distance to the visible platform in the second than in the first trial and spent more time in the correct quadrant. There was a significant diet×sex interaction for latency (*P*=0.04; Fig. 1A-C) and cumulative distance to the platform (*P*=0.02; Fig. 2A-C), as XN improved performance in females but not in males. The effect was significant in XN-supplemented versus control wild-type females (*P*=0.02 for latency and *P*=0.006 for cumulative distance to the platform), but not in FXR mutant females. Performance in FXR^Liver−/−^ mice was better than in FXR^Intestine−/−^ mice for both latency (*P*=0.02) and cumulative distance to the platform (*P*=0.03), with intermediate values for wild-type mice (Fig. 1D-I, Fig. 2D-I, Fig. 3D-I). No sex effect was observed for latency or cumulative distance to the platform. Female mice spent more time in the correct quadrant (i.e. the quadrant containing the platform) than males (*P*=0.009; Fig. 3A-C). No effect of XN or genotype was observed for time spent in the correct quadrant.

### XN supplementation improves acquisition during hidden platform trials

Acquisition improved during the six hidden platform trials, as mice reached the platform faster, with a lower cumulative distance to the hidden platform with each trial, and spent more time in the correct quadrant. There were effects of genotype and sex on latency and cumulative distance to the platform in the hidden platform trials; females reached the hidden platform faster (*P=*0.02) and with a lower cumulative distance to the platform (*P=*0.01) than males, and FXR^Liver−/−^ and wild-type mice reached the platform faster (*P=*0.005) and with a lower cumulative distance to the platform (*P=*0.01) than FXR^Intestine−/−^ mice. In all six genotype×sex combinations, XN-treated mice had a lower cumulative distance to the hidden platform in the first trial than control mice. In all six genotype×sex combinations, XN-supplemented mice spent on average more time in the correct quadrant than control mice. No effect of sex or genotype was observed for time spent in the correct quadrant.

### XN supplementation improves reversal learning of males

Reversal learning improved during the two reversal platform trials, as mice reached the hidden platform in the new location faster and with a lower cumulative distance to the hidden platform in the second than in the first trial, and they spent more time in the correct quadrant (Figs 1-3). Supplementation with XN restored all three performance measures of reversal learning in control genotype/sex combination groups with relatively poor performance, which were usually males. Supplementation with XN restored reversal learning in males, with the strongest impact in FXR^Intestine−/−^ males and a significant impact in wild-type males as well. The only of the six genotype×sex combinations with poorer performance in XN supplemented versus control mice were FXR^Intestine−/−^ females.

### XN supplementation improves some aspects of spatial memory retention

Spatial memory retention was assessed in the probe trial (no platform). Cumulative distance to the platform location was used as one performance measure. Differences in performance were observed for the first and last 30 s of the probe trial. In the first 30 s of the probe trial, female FXR^Liver−/−^ mice had a lower cumulative distance to the platform location than male FXR^Liver−/−^ mice (*P=*0.0003). In the last 30 s of the probe trial, XN-supplemented mice in all six genotype×sex combinations on average had a higher cumulative distance to the platform location than control mice (Fig. S1).

**Fig. 4.**
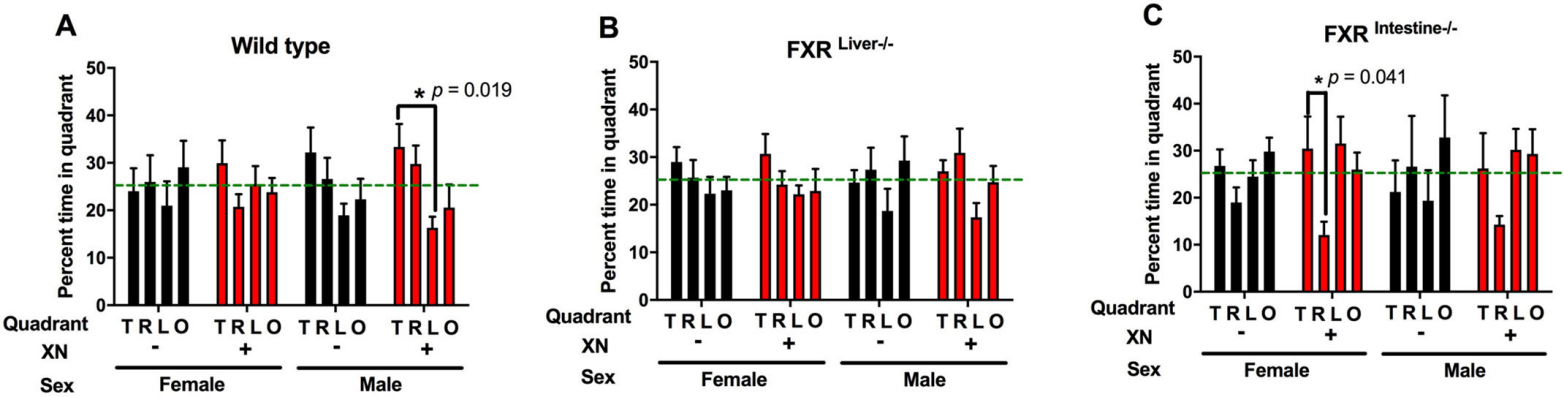
**Percentage of time spent in opposite (O), right (R), target (T) and left (L) quadrants in the probe trial.** The target quadrant is the quadrant most recently containing the hidden platform. A higher percentage of time spent in the target quadrant indicates better performance. In male wild-type and female FXR^Intestine−/−^ mice, XN-treated mice spent more time in the target quadrant than in the left or right quadrant, respectively.

We also assessed performance in the probe trial by analyzing the percentage of time spent by mice in the target quadrant, which contained the platform during the hidden platform training, compared to the three non-target quadrants. In all 12 XN/genotype/sex combinations, mice spent more time in the opposite quadrant (*P=*0.0005) and less time in the other two non-target quadrants (*P=*0.04) in the first 30 s than in the last 30 s (Fig. 4). There were no effects of XN supplementation, genotype or sex on the percentage of time spent in the target quadrant in the probe trial.

### XN alters diacylglycerol levels in a genotype- and sex-dependent manner

Supplementation with XN affected diacylglycerol (DAG) levels differently based on sex and genotype (Tables S1-S3). XN-supplemented female FXR^Liver−/−^ mice had higher levels of 31 of 40 individual DAGs than control female FXR^Liver−/−^ mice. In contrast, XN-supplemented male FXR^Liver−/−^ mice had lower levels of 29 of 40 individual DAGs than control male FXR^Liver−/−^ mice (*P*<0.0001). FXR^Liver−/−^ mice had higher total DAG levels (*P=*0.04) and higher levels of 12 of 40 individual DAGs than FXR^Intestine−/−^ mice, while wild-type mice had intermediate DAG levels. Females mice had higher (*P<*0.05) levels of six individual DAGs (C36:1, C38:1, C38:2, C38:4, C38:5, C42:7) and tended to have higher (0.10<*P<*0.05) levels of four individual DAGs (C38:0, C38:3, C38:4, C40:6) than male mice.

### XN increases the ratio of short-chain to long-chain fatty acids in ceramides and hexaceramides

Supplementation with XN altered the ratio of long-chain to short-chain fatty acids in ceramides without affecting total ceramide concentrations (Tables S4-S6). Specifically, XN supplementation increased the levels of ceramides and hexaceramides of carbon chain length 34 and shorter (XN mice had higher values than their control counterparts in 17 of 19 genotype×sex combinations) and decreased the levels of ceramides and hexaceramides of carbon chain length 38 and longer (XN mice had lower values than their control counterparts in 51 of 54 genotype×sex combinations).

### XN alters sphingomyelin levels in a genotype- and sex-dependent manner

XN supplementation affected sphingomyelin and sphinganine levels differently based on sex and genotype (Tables S7-S9). XN-supplemented FXR^Intestine−/−^ male mice had lower levels of 24 of 25 individual sphingomyelins than control FXR^Intestine−/−^ male mice (*P<*0.0001). In contrast, XN-supplemented female mice (17 for wild-type mice, 19 for FXR^Liver−/−^ mice, 16 for FXR^Intestine−/−^ mice) had higher levels of 18 of 25 individual sphingomyelins than control female mice (*P=*0.04). No consistent sex×genotype interactions were observed for individual and total sphingomyelin levels. There was a significant genotype×sex×XN supplementation interaction (*P=*0.01) for total sphinganine levels, with higher levels in XN-supplemented versus control wild-type males (*P=*0.02). Sphinganine levels were also higher in XN-supplemented versus control FXR^Liver−/−^ females (*P=*0.005).

### Lower levels of sphinganine and longer-chain ceramide and hexaceramide levels are linked to improved visible platform learning

Ceramides and sphingosines and sphinganine, but not DAG and sphingomyelins, were correlated with performance in the visible platform trials. Five of 12 performance measures (42%) of the visible platform trials were correlated with total sphinganine levels (*P<*0.05). Three of 12 performance measures (25%) of the visible platform trials were correlated with total ceramide and total hexaceramide levels (both *P<*0.05). More significant correlations were observed for the longer-chain ceramides and hexaceramides than for the shorter-chain ceramides.

### Higher levels of DAG and lower levels of longer-chain ceramides and hexaceramides are linked to improved hidden platform learning

A high proportion of DAG and ceramides, but not sphingomyelins, was correlated with performance in the hidden platform trials. Eleven of 27 performance measures (41%) of the hidden platform trial were positively correlated with total ceramide and total hexaceramide levels (both *P<*0.05). Six of 27 measures (22%) of the hidden platform trials were negatively correlated with total DAG levels (*P<*0.05). Two of the 27 measures (7%) of the hidden platform trial were negatively correlated with total sphingomyelin (*P<*0.05). The primary correlations were with latency and cumulative distance to the hidden platform. Lower total ceramide and hexaceramide levels were linked to improved performance, lower latency (*r*=+0.37; *P=*0.0003 and *r*=+0.39; *P=*0.0001) and lower cumulative distance to the platform (*r*=+0.32; *P=*0.002 and *r=*+0.33; *P=*0.001). More significant correlations were observed for the longer-chain ceramides and hexaceramides than for the shorter-chain ceramides. Higher total DAG levels were linked to shorter latency (*r*=−0.23; *P=*0.02) and cumulative distance to the platform (*r*=−0.27; *P=*0.02).

### Lower levels of longer-chain ceramides and hexaceramides are linked to improved reversal platform learning

Levels of ceramides, but not DAGs and sphingomyelins, were correlated with performance in the reversal platform trials. Three of nine measures (33%) of the reversal platform trials were correlated with total ceramide and total hexaceramide levels (*P<*0.05). Total DAG, total sphingomyelin, sphingosine and sphinganine were not correlated with performance in the reversal platform trials. Lower total ceramide and hexaceramide levels were linked to lower latency (*r*=+0.22; *P=*0.01 and *r=*+0.21; *P=*0.01), lower cumulative distance to the platform (*r*=+0.17; *P=*0.04 and *r*=+0.16; *P=*0.05) and more time spent in the correct quadrant (*r*=−0.26; *P=*0.10 and *r*=−0.26; *P=*0.13). More significant correlations were observed for the longer-chain ceramides and hexaceramides than for the shorter-chain ceramides.

### Higher levels of DAG and lower levels of longer-chain ceramides and hexaceramides are linked to improved performance in the probe trial

Ceramides and DAG, but not sphingomyelins, were correlated with performance in the probe trial. Five of 15 measures (33%) of the probe trial were correlated with total DAG and total ceramide levels (both at *P<*0.05). Four of 15 measures (27%) of the probe trial were correlated with total hexaceramide levels, while no measures were correlated with total sphingomyelin levels. Higher total DAG levels were linked to more time spent in the correct quadrant (*r*=+0.28; *P=*0.008) and a lower cumulative distance to the platform (*r*=−0.31; *P=*0.03) in the first 30 s of the trial. Higher total ceramide and hexaceramide levels were linked to less time spent in the correct quadrant in the first 30 s of the trial (r=−0.22; *P=*0.04 and r=−0.23; *P=*0.03) but more time spent in the correct quadrant in the last 30 s of the probe trial (*r*=+0.24; *P=*0.02 and *r*=+0.25; *P=*0.02).

## DISCUSSION

The data of this study show that, in wild-type, FXR^Intestine−/−^ and FXR^Liver−/−^ mice, XN improves cognitive performance and alters hippocampal DAG and sphingomyelin levels in a genotype- and sex-dependent manner. XN improves task learning in females (specifically wild-type), reversal learning in males (specifically wild-type and FXR^Intestine−/−^ mutant) and spatial learning in both sexes. Whereas XN increases hippocampal DAG and sphingomyelin levels in females, XN decreases hippocampal DAG and sphingomyelin levels in males. XN increases the ratio of shorter-chain to longer-chain ceramides and hexaceramides. Higher levels of DAG and lower levels of longer-chain ceramides and hexaceramides levels are linked to improved cognitive performance. Thus, the beneficial sex-dependent cognitive effects of XN are linked to changes in hippocampal DAG and ceramide levels.

FXR plays an important role in the regulation of lipids and glucose, as well as bile acid homeostasis (Paraiso et al., 2021). Overaccumulation of bile acids and lipids can lead to liver damage (Paraiso et al., 2021). Bile acids act in a negative-feedback loop to inhibit their own oversynthesis by acting mainly through FXR (Goodwin et al., 2000). We previously reported that XN protected FXR^Liver−/−^ mice from HFD-induced liver damage and changed bile acid composition in a genotype-dependent manner (Paraiso et al., 2021). XN also improved HFD-induced dysfunctional lipid and bile acid metabolism via FXR-dependent and -independent signaling (Goodwin et al., 2000). In the present study, the largest effects of XN on water maze performance were seen during visible platform training (a measure of cue-based task learning) and reversal learning (a measure of cognitive flexibility). Some of the cognitive effects were sex dependent. During visible platform trials, XN improved performance in females, but impaired performance in males. During reversal learning, XN improved performance in both sexes in wild-type mice, although more dramatically in males than females. In FXR^Intestine−/−^ mice, XN impaired performance in females, but dramatically improved reversal learning in males. XN-treated FXR^Intestine−/−^ males also showed markedly better performance in the last two sessions of hidden platform training compared to HFD-only males. In contrast, aside from during session 8, XN did not affect any cognitive performance in FXR^Liver−/−^ mice. These data suggest that FXR in the liver is important in mediating the cognitive effects of XN. FXR in the liver or elsewhere outside the intestine might also be important in mediating the beneficial effects of XN on spatial leaning and reversal learning in FXR^Intestine−/−^ male mice. In addition, in the intestine, XN might have beneficial effects on cognitive performance via a mitochondrial uncoupling effect independent of FXR. XN increases uncoupled respiration, induces oxidant defense mechanisms, and lowers reactive oxygen species (ROS) and dysfunctional lipid metabolism (Kirkwood et al., 2013; Stevens, 2020; Stevens et al., 2018).

In wild-type mice, XN improved the ability of female, but not male, mice to locate the visible platform. This sex difference might be related to the metabolic transformation of XN into the metabolite 8PN, which has estrogenic activity (Milligan et al., 1999, 2000; Miranda et al., 2018; Possemiers et al., 2006).

**Fig. 5.**
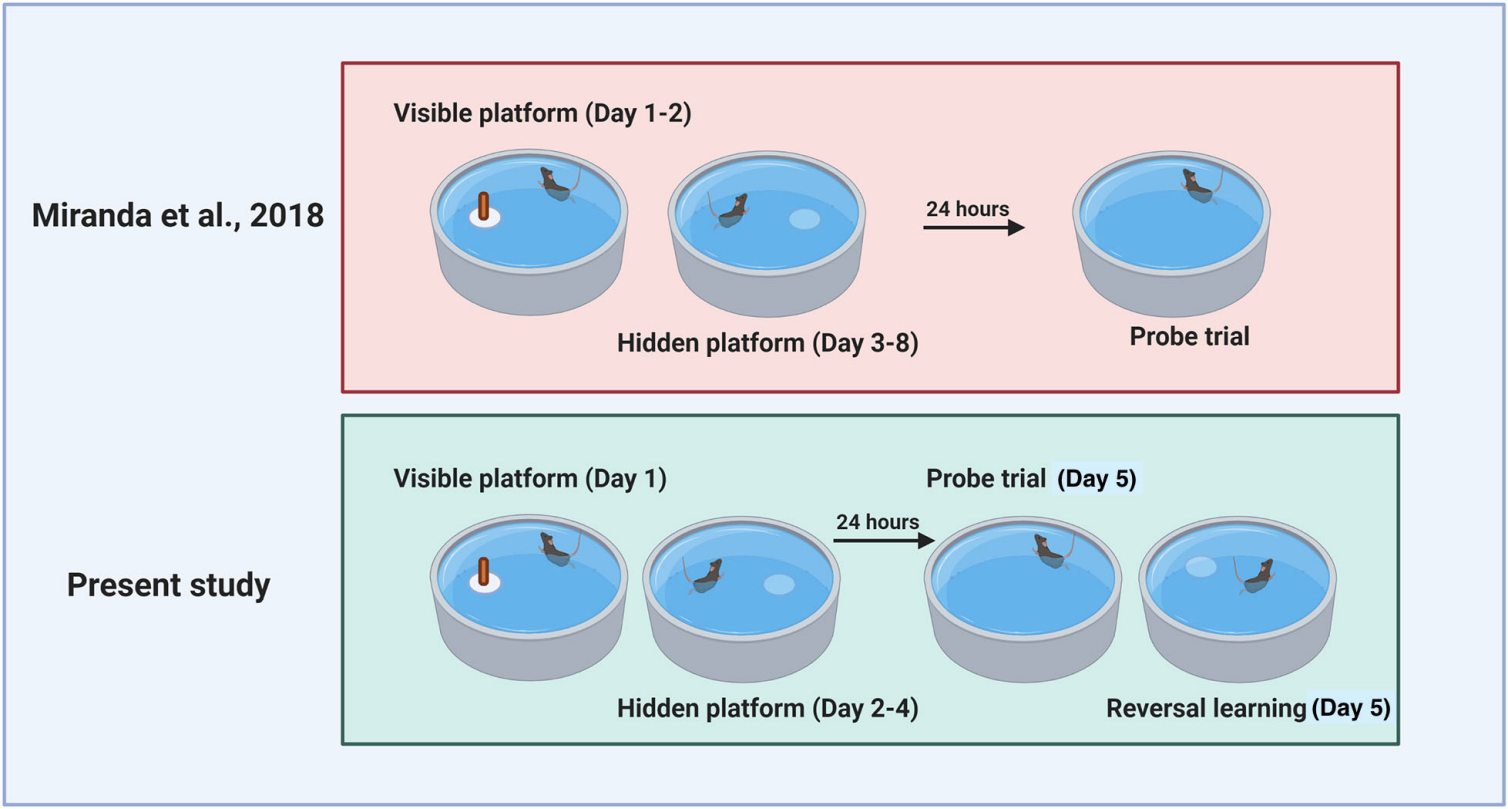
**Water maze design used in our current and previous studies.** Comparison of the water maze paradigms used in our current study (bottom) and our previous study (top; Miranda et al., 2018). Figure created using Biorender.com software.

Previously, we reported that 13 weeks of treatment with XN ameliorates HFD-induced deficits in spatial learning in a water maze (Miranda et al., 2018). In contrast to the present study, the time to locate the hidden platform was lower in XN-supplemented mice than in HFD-only mice (Miranda et al., 2018). This discrepancy might be due to the longer length of training in the previous study (6 days of hidden platform) than in the present study (3 days). A comparison of the water maze paradigms used in the two studies is shown in Fig. 5. Indeed, the largest treatment effect of XN on time to locate the hidden platform in the previous study was observed in the latter 3 days of training. Thus, it is possible that treatment effects of XN on performance during hidden platform training could have been revealed in the current study if testing had been conducted for additional days. In a probe trial administered 24 h after the last hidden platform trial, XN improved the latency to first cross the previous platform location, an effect not seen in the present study. Again, it is possible that with the longer training to locate the hidden platform location prior to the probe trial, group differences could have emerged previously that were not seen in this study.

In the current study, lower levels of longer-chain ceramides and hexaceramides were linked to improved cognitive performance. This result is consistent with declining verbal memory over time in patients with coronary artery disease with increased baseline plasma ceramide C18:0 levels (Chan et al., 2018), and higher baseline plasma ceramide C22:0 and C24:0 levels predicting less improvement in verbal memory performance over 1 year of cardiac rehabilitation (Saleem et al., 2013). In addition, plasma ceramides C22:0 and C24:0 predict memory loss and right hippocampal volume loss in patients with mild cognitive impairments (Mielke et al., 2010). Further, elevated ceramide levels appear to worsen leptin resistance, which is important in the pathophysiology of obesity and metabolic syndrome (Field et al., 2020). Secretion of long-chain ceramides C40:1 and C42:1 was also shown to be involved in chronic endoplasmic reticulum stress in skeletal muscle, linking lipotoxicity to metabolic disease (McNally et al., 2022).

The hippocampal lipid pattern in the current HFD study is also consistent with spatial reference memory [(first visit+revisits of baited holes)/total visits of all holes] in a hole board test and the hypothalamic lipid pattern in a cognitive aging study involving young (3-month-old) and old (20-month-old) male rats (Wackerlig et al., 2020). Two of seven ceramides (C17:2 and C19:2) were higher in old cognitively impaired rats than in young cognitively unimpaired rats. In addition, ceramide C17:2 was higher in old cognitively impaired rats than in old cognitively unimpaired rats. Further, although four of 13 hexosyceramides (HexCer), derivatives of ceramides, all containing short and mostly saturated chains (HexCer 18:0, 22:0, 22:1 and 24:0) were lower in old cognitively impaired rats than in young cognitively unimpaired rats, HexCer 26:4, which possesses a longer polyunsaturated chain, was higher in old cognitively impaired rats than in young cognitively impaired rats. For cognitive performance, HexCer 22:0 and 24:0 seemed most important, as HexCer 18:0 and 22:1 were also lower in cognitively unimpaired old rats than in young cognitively unimpaired rats. These data indicate possible overlap in the role of ceramides in cognitive aging and cognition under conditions of an HFD in young adults. Because the two brain regions analyzed in these two studies were distinct, these data suggest that the ceramide patterns might reflect global changes in brain and not show anatomical specificity. Further studies are warranted to determine whether, in the context of an HFD, this ceramide pattern is also seen in brain regions other than the hippocampus and whether brain ceramide levels are associated with cognitive performance.

However, in contrast to the current study and the studies discussed above, higher plasma ratios of ceramides C24:0/C16:0 and C22:0/C16:0 were associated with reduced dementia risk and a lower white matter hyperintensity burden in magnetic resonance imaging in the Framingham study community-based sample (McGrath et al., 2020). Sex and the apolipoprotein E (APOE) isoform APOE4, a risk factor for developing Alzheimer's disease (Farrer et al., 1997), might contribute to these divergent data. Although in men, the highest tertile of most ceramides and sphingomyelins were associated with an increased risk of developing Alzheimer's disease, in women, there were no associations between any of the ceramides and risk of developing Alzheimer's disease, and in women with the highest tertile of most sphingomyelins there was a reduced risk of developing Alzheimer's disease, which was most pronounced among those carrying APOE4 (Mielle et al., 2017). Sex differences in hippocampal lipid pattern following treatment with XN were also seen in the current study. XN increased hippocampal DAG and sphingomyelin levels in females, but decreased them in males. XN increased the ratio of shorter-chain to longer-chain ceramides and hexaceramides. Thus, beneficial sex-dependent cognitive effects of XN are linked to changes in hippocampal DAG and ceramide levels. These data highlight the importance of including both sexes in assessing the effects of XN in cognitive studies.

When spatial memory retention in the probe trial was assessed, performance was better in the first than in the last 30 s. It is conceivable that after 30 s of not being able to locate the platform the animals search for it elsewhere. Intriguingly, higher total ceramide and hexaceramide levels were linked to less time spent in the correct quadrant in the first 30 s of the trial, but more time spent in the correct quadrant in the last 30 s of the probe trial. Thus, lower ceramide and hexaceramide levels seem especially important for improved cognitive performance under conditions of an HFD.

In this study and our previous study (Miranda et al., 2018), the mice were 2-3 months old at the beginning of the study prior to the HFD and XN treatment. Recently, we showed that XN is also beneficial in treating HFD-induced cognitive impairments in mice that are 6 months old when treated with an HFD and XN (Kundu et al., 2022). APOE is involved in lipid metabolism and distribution, and, compared to APOE3, APOE4 increases the risk of developing age-related cognitive decline and Alzheimer's disease (Farrer et al., 1997; Raber et al., 2004). Therefore, in addition to wild-type mice, mice expressing human APOE3 and APOE4 were included. XN was associated with sex- and APOE isoform-dependent effects on cognitive performance (Kundu et al., 2022). XN-treated APOE4-expressing and wild-type, but not APOE3-expressing, mice had higher glucose transporter protein levels in the hippocampus and cortex than HFD-treated mice. Higher glucose transporter protein levels were linked to better fear learning and cued fear memory and better spatial learning and memory in the water maze (Kundu et al., 2022), suggesting that alterations to glucose transporter protein levels might mediate some beneficial cognitive effects of XN. XN has also been shown to improve cognitive performance and reduce neuropathology in 9-month-old male mice with transgenic expression of human amyloid precursor protein and presinilin 1 with familiar Alzheimer's disease mutations (Sun et al., 2021). In addition, XN increases superoxide dismutase levels in the hippocampus and serum (Sun et al., 2021), consistent with XN promoting the antioxidant defense mechanisms described earlier (Kirkwood et al., 2013; Stevens, 2020; Stevens et al., 2018).

**Fig. 6.**
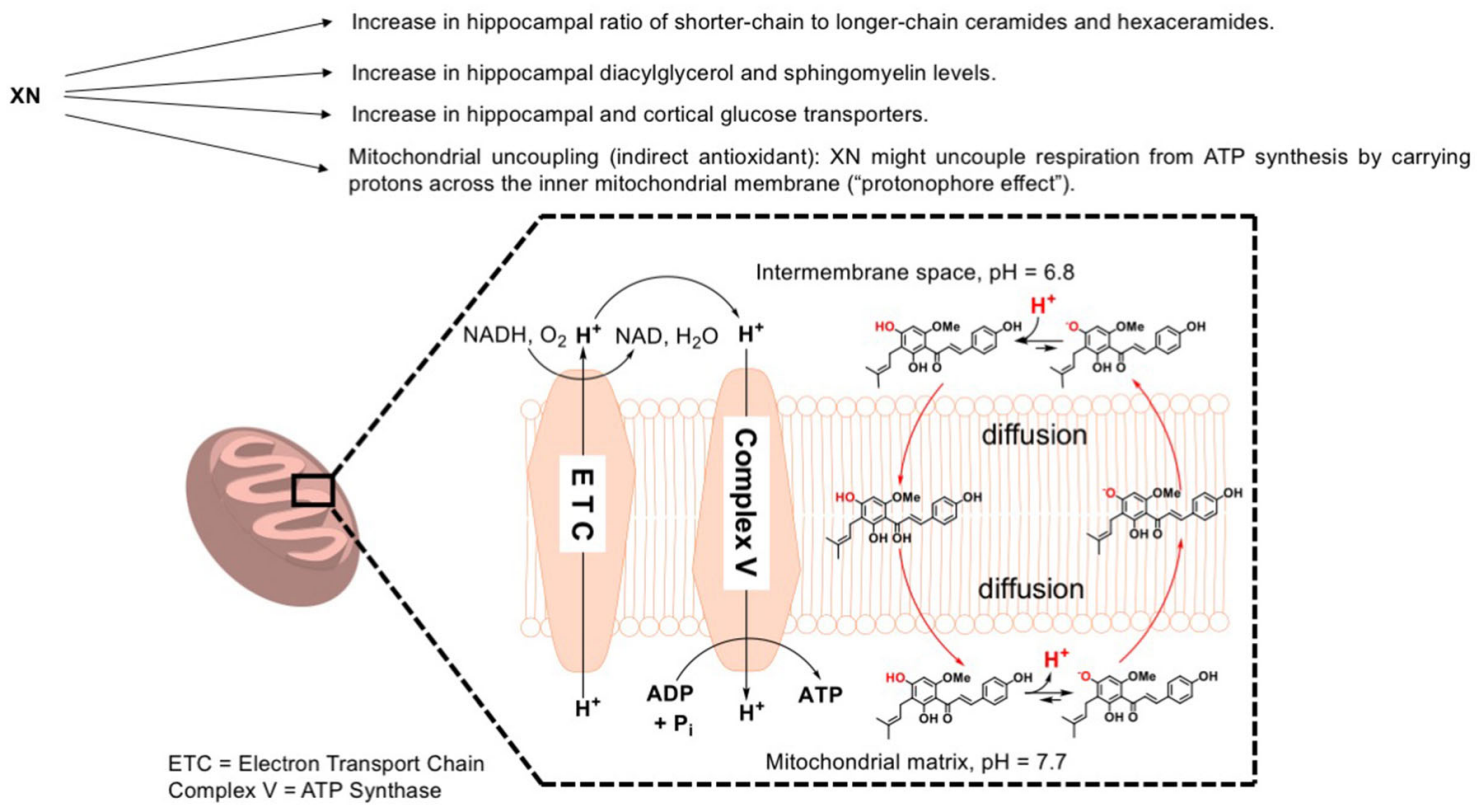
**Summary of possible mechanisms underlying the beneficial cognitive effects of XN.** Effects of XN that micht contribute to its beneficial cognitive effects are indicated by the arrows in the top part of this figure. The bottom part illustrates mitochondrial function that might be involved in these beneficial effects of XN on cognition.

In summary, the data of the current study show that higher levels of DAG and lower levels of longer-chain ceramides and hexaceramides are associated with improved cognitive performance. Under conditions of an HFD in young mice, the beneficial cognitive effects of XN are sex dependent and linked to changes in hippocampal DAG and ceramide levels. Fig. 6 summarizes the possible mechanisms underlying the beneficial cognitive effects of XN. Further efforts to assess the effects of XN on age-related cognitive decline and cognitive impairments in animal models of neurodegeneration, and to determine whether these effects are associated with changes in lipid profiles in pertinent brain regions, are warranted.

## MATERIALS AND METHODS

### Animals and treatment

Animals were treated as previously described (Paraiso et al., 2021). All animal experiments were performed in accordance with institutional and National Health and Medical Research Council guidelines and followed Animal Research: Reporting of *In Vivo* Experiments (ARRIVE) guidelines. The experimental protocol was approved by the Institutional Animal Care and Use Committee (IACUC) at Oregon State University, and the studies were carried out in accordance with the approved protocol (IACUC 2019-0001). Briefly, wild-type male and female C57BL/6J mice at the age of 9 weeks were obtained from The Jackson Laboratory (Bar Harbor, ME, USA). FXR^Liver−/−^ and FXR^Intestine−/−^ mice were generated on a C57BL/6J using a cre recombinase system. Mice were housed in groups of two to three in ventilated cages and were on a 12:12 h light–dark cycle. All mice were fed an HFD (Dyets Inc., Bethlehem, PA, USA) containing 60%, 20% and 20% total calories from fat, carbohydrate and protein, respectively. XN was mixed into the diet of a subset of mice to deliver an approximate dose of 60 mg/kg body weight/day as previously described (Miranda et al., 2018). Mice were treated with XN or a vehicle control HFD for 12 weeks prior to behavioral testing. After the conclusion of behavioral testing, mice were euthanized by cervical dislocation, and blood, hippocampus and cortex were collected and stored at −80°C for further analysis. The researcher behaviorally testing the mice was blinded to the genotype and treatment. The code was broken once all the data had been acquired and analyzed.

### Hippocampal lipid analysis

Hippocampal lipids were extracted as described (Paraiso et al., 2020) using a one-phase solvent system (500 μl, 25:10:65 v/v/v system of methylene chloride: isopropanol: methanol, with 50 μg/ml butylated hydroxytoluene) with some modification. Hippocampal samples (≈10 mg, *n*=9-12 per group) were homogenized with 0.5 mm zirconium oxide beads (Next Advance Inc., Troy, NY, USA) using a counter-top bullet blender for 3 min and then incubated at −20°C for 1 h, and the homogenates were centrifuged at 15,000 ***g*** at 4°C for 10 min. From each extract, a 40 μl aliquot from the supernatant was transferred to an autosampler vial, and 155 μl extraction solvent and 5 μl Splash Lipidomix Mass Spec Standard (Avanti Polar Lipids, Alabaster, AL, USA) were added. Samples were stored at −80°C until further analysis. Liquid chromatography and mass spectrometry conditions were developed as described previously (Paraiso et al., 2021). Peak alignment, feature extraction and normalization were performed using Progenesis QI (Waters, Milford, MA, USA). The area of the base peak extracted ion chromatogram was selected for relative quantitation.

### Behavioral and cognitive analyses

#### Spatial learning and memory in the water maze

The water maze task was conducted using a circular pool (diameter, 129 cm), filled with water (22°C). White chalk was added to the water to make it opaque. The maze was divided conceptually into four quadrants. Extra-maze cues of various shapes, sizes and colors were placed around the maze. A white partition separated the maze from the rest of the room during testing. This was done to isolate the maze and extra-maze cues from the surrounding room, as well as from the experimenter. Mice were tested for two sessions per day (separated by 3 h), with each session consisting of two trials (separated by 10 min). Testing was conducted over the course of 5 days: 1 day of visible platform training, 3 days of hidden platform training, followed by a final day consisting of a probe trial (no platform), followed by two hidden platform reversal trials in which the hidden platform was moved to a new location. Mice were first trained to locate a submerged circular Plexiglas platform (diameter, 12 cm) 2 cm below the surface of the water. During the visible platform trials, the platform was marked by the use of a black cue flag. Once mice found the platform and remained on it for 3 s, they were removed from the pool and returned to their home cage. Mice were placed into the maze from various starting locations at the beginning of each visible platform trial to avoid any potential bias. Following the two sessions of visible platform training, the cue was removed and hidden platform training began. During hidden platform trials, the platform remained in one location. Spatial memory retention was assessed with a probe trial after 3 days (six sessions) of hidden platform training. During probe trials, the platform was removed from the pool and mice remained in the pool for the full 60 s trial. Following the probe trial, the platform was placed back into the pool in a new hidden location for two more training sessions. Performance of the mice was recorded using AnyMaze software (Stoelting, Wood Dale, IL, USA) and analyzed using EthoVision software (Version 7.0 XT; Noldus, Leiden, The Netherlands).

#### Statistical analyses

Data are expressed as mean±s.e.m. Graphs were generated using Prism software (Version 8.2.0; GraphPad, La Jolla, CA, USA). Data were analyzed using SPSS Statistics for Windows (Version 25; IBM Corp., Armonk, NY, USA) and SAS (Version 9.4; SAS Institute Inc., Cary, NC, USA). Cognitive and lipidomic measures were analyzed using one-, two- and three-way ANOVA or repeated measures ANOVA. The fixed effects were dietary treatment (HFD or XN), genotype (wild-type, FXR^Intestine−/−^ and FXR^Liver−/−^, APOE3-expressing or APOE4-expressing), sex (female or male) and their two- and three-way interactions. Given the complex experimental design, we also examined the treatment effect for each genotype×sex×treatment exposure separately for a total of six comparisons. Main effects of treatment were determined using a sign test by calculating the number of positive and negative differences across the six genotype×sex×treatment exposure length combinations. Statistical significance was determined using an error probability level of *P*<0.05.

Correlations between cognitive and lipidomic measures were analyzed using the non-parametric Spearman rank test. To account for multiple comparisons and the false discovery rate, we report only on lipidomic/metabolomics/glucose transporter measures that had at least 20% of functional behavioral measures significantly correlated at *P*<0.05.

## Supplementary Material

10.1242/dmm.049820_sup1 Supplementary information
