# Supplementary material for: Xanthohumol improves cognition in farnesoid X receptor-deficient mice on a high-fat diet
Source: Dis Model Mech. 2022 Nov 25;15(11):dmm049820. doi: 10.1242/dmm.049820 (PMC9713832; doi:10.1242/dmm.049820)
Supplement: Supplementary information [file dmm-15-049820-s1.pdf]

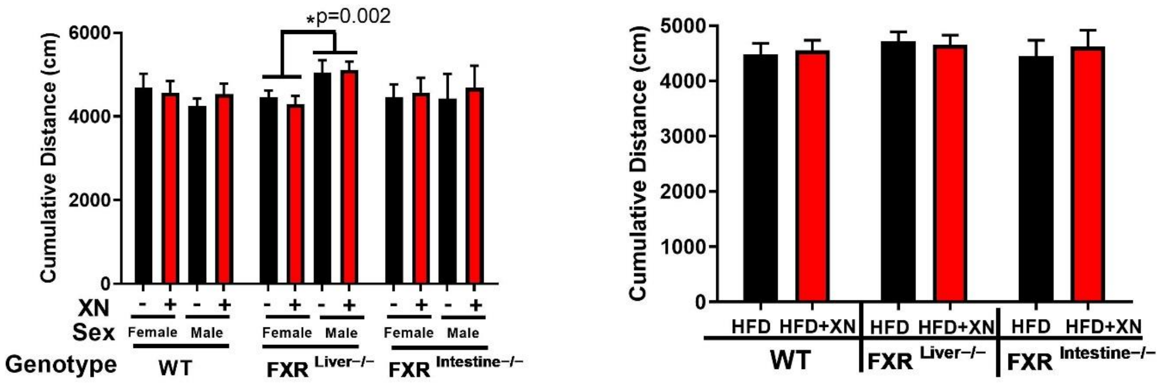

Fig. S1. Cumulative distance to previous platform location in the probe trial. Data is presented with sexes separate **(left)** as well as combined **(right)**. Mice that received a high fat diet alone are indicated with **(-)** and mice that received a high fat diet combined with XN are indicated with **(+)**. In $FXR^{Liver-/-}$ mice, females showed better performance than males. There were no effects of diet in any genotype.

Disease Models & Mechanisms • Supplementary information

Table S1. Diacylglycerol levels affected by XN in the hippocampus of wild-type mice.

	Female					Male				
Wild-type	HFD		XN		*P*-value	HFD		XN		*P*-value
Hippocampus	Mean	SEM	Mean	SEM		Mean	SEM	Mean	SEM	
DAG 30:0	0.008	0.001	0.008	0.002	0.96	0.007	0.001	0.006	0.001	0.54
DAG 30:1	0.003	0.000	0.004	0.000	0.45	0.003	0.001	0.003	0.001	0.93
DAG 30:2	0.007	0.001	0.010	0.001	0.17	0.009	0.002	0.008	0.002	0.60
DAG 32:0	0.138	0.015	0.132	0.014	0.74	0.140	0.016	0.122	0.011	0.33
DAG 32:1	0.435	0.068	0.416	0.051	0.77	0.405	0.047	0.389	0.032	0.81
DAG 32:2	0.007	0.001	0.007	0.001	0.70	0.006	0.001	0.007	0.001	0.75
DAG 32:3	0.006	0.001	0.007	0.001	0.26	0.007	0.000	0.008	0.001	0.51
DAG 34:0	0.534	0.072	0.547	0.067	0.88	0.502	0.063	0.513	0.044	0.91
DAG 34:1	1.979	0.290	1.995	0.253	0.96	1.902	0.198	1.807	0.153	0.77
DAG 34:2	1.277	0.189	1.206	0.144	0.72	1.187	0.136	1.080	0.085	0.62
DAG 34:3	3.928	0.562	3.818	0.543	0.86	3.682	0.416	3.411	0.231	0.69
DAG 36:1	1.099	0.142	0.677	0.050	0.03	0.693	0.081	0.515	0.079	0.37
DAG 36:2	2.306	0.311	2.256	0.222	0.89	2.292	0.252	2.125	0.168	0.67
DAG 36:3	5.763	0.702	5.579	0.733	0.84	5.843	0.817	5.207	0.638	0.51
DAG 36:4	2.044	0.312	1.968	0.187	0.81	1.986	0.227	1.700	0.109	0.39
DAG 36:5	0.035	0.007	0.034	0.005	0.90	0.033	0.003	0.029	0.003	0.90
DAG 36:6	0.039	0.005	0.051	0.007	0.11	0.047	0.005	0.047	0.006	0.97
DAG 38:0	0.088	0.013	0.064	0.006	0.06	0.078	0.011	0.057	0.007	0.14
DAG 38:1	0.102	0.012	0.058	0.003	**0.004**	0.067	0.006	0.045	0.010	0.17
DAG 38:2	0.111	0.016	0.077	0.005	0.08	0.075	0.009	0.063	0.009	0.55
DAG 38:3	0.374	0.052	0.312	0.017	0.36	0.324	0.031	0.261	0.037	0.38
DAG 38:4	5.757	0.732	5.209	0.304	0.56	5.254	0.465	4.373	0.540	0.38
DAG 38:5	0.494	0.063	0.400	0.032	0.21	0.450	0.035	0.341	0.029	0.17

Disease Models & Mechanisms • Supplementary information

DAG 38:6	0.494	0.064	0.484	0.032	0.90	0.476	0.061	0.428	0.048	0.56
DAG 38:7	0.057	0.007	0.061	0.007	0.64	0.057	0.005	0.061	0.007	0.62
DAG 38:8	0.089	0.011	0.096	0.012	0.55	0.098	0.012	0.102	0.005	0.74
DAG 40:4	0.241	0.038	0.191	0.038	0.30	0.170	0.035	0.196	0.043	0.62
DAG 40:5	0.042	0.004	0.038	0.004	0.42	0.042	0.005	0.037	0.003	0.36
DAG 40:6	0.240	0.027	0.211	0.009	0.45	0.239	0.016	0.204	0.021	0.41
DAG 40:7	0.091	0.013	0.086	0.011	0.67	0.088	0.009	0.084	0.009	0.78
DAG 40:8	0.036	0.004	0.036	0.004	0.97	0.035	0.003	0.032	0.005	0.58
DAG 42:0	0.001	0.000	0.002	0.000	0.22	0.001	0.000	0.003	0.001	**0.02**
DAG 42:1	0.020	0.003	0.013	0.002	0.07	0.016	0.002	0.012	0.003	0.29
DAG 42:2	0.014	0.003	0.011	0.002	0.27	0.013	0.001	0.010	0.002	0.38
DAG 42:6	0.672	0.103	0.632	0.072	0.70	0.677	0.105	0.642	0.069	0.76
DAG 42:7	0.411	0.015	0.425	0.029	0.59	0.382	0.028	0.412	0.017	0.30
DAG 44:0	0.007	0.001	0.009	0.002	0.20	0.009	0.001	0.008	0.001	0.54
DAG 44:1	0.002	0.000	0.002	0.001	0.53	0.002	0.000	0.003	0.001	0.04
DAG 44:2	0.025	0.003	0.023	0.003	0.59	0.023	0.004	0.022	0.004	0.82
DAG 44:8	0.285	0.018	0.291	0.021	0.86	0.269	0.028	0.271	0.031	0.94
Total DAG	29.263	3.628	27.445	2.186	0.63	27.588	2.788	24.640	1.856	0.47

Disease Models & Mechanisms • Supplementary information

Table S2. Diacylglycerol levels affected by XN in the hippocampus of FXR$^{Liver-/-}$ mice.

	Female					Male				
FXR$^{Liver-/-}$	HFD		XN		*P*-value	HFD		XN		*P*-value
Hippocampus	Mean	SEM	Mean	SEM		Mean	SEM	Mean	SEM	
DAG 30:0	0.007	0.001	0.010	0.001	0.13	0.008	0.002	0.009	0.001	0.41
DAG 30:1	0.003	0.000	0.004	0.000	0.14	0.004	0.000	0.003	0.000	0.40
DAG 30:2	0.008	0.001	0.008	0.001	0.96	0.009	0.001	0.008	0.001	0.84
DAG 32:0	0.121	0.014	0.147	0.014	0.11	0.130	0.010	0.120	0.008	0.57
DAG 32:1	0.454	0.037	0.466	0.033	0.83	0.419	0.053	0.438	0.045	0.77
DAG 32:2	0.006	0.001	0.008	0.001	0.21	0.007	0.001	0.007	0.001	0.98
DAG 32:3	0.008	0.001	0.007	0.001	0.83	0.007	0.001	0.006	0.001	0.38
DAG 34:0	0.593	0.053	0.608	0.054	0.85	0.597	0.086	0.562	0.059	0.69
DAG 34:1	2.281	0.219	2.304	0.165	0.93	2.109	0.274	2.075	0.201	0.91
DAG 34:2	1.274	0.153	1.239	0.133	0.85	1.191	0.162	1.143	0.111	0.81
DAG 34:3	4.142	0.454	4.404	0.452	0.65	3.862	0.530	4.255	0.379	0.54
DAG 36:1	0.998	0.232	1.060	0.139	0.71	0.632	0.037	0.765	0.099	0.48
DAG 36:2	2.771	0.276	2.650	0.207	0.81	2.440	0.309	2.396	0.271	0.90
DAG 36:3	6.730	0.541	6.108	0.537	0.44	5.646	0.611	6.006	0.822	0.69
DAG 36:4	2.202	0.216	2.318	0.229	0.68	2.009	0.253	2.109	0.179	0.75
DAG 36:5	0.039	0.004	0.035	0.004	0.50	0.043	0.006	0.034	0.003	0.21
DAG 36:6	0.046	0.003	0.039	0.004	0.37	0.050	0.007	0.041	0.003	0.24
DAG 38:0	0.074	0.007	0.084	0.009	0.37	0.062	0.009	0.065	0.009	0.84
DAG 38:1	0.080	0.014	0.112	0.010	**0.02**	0.075	0.008	0.082	0.012	0.62
DAG 38:2	0.100	0.023	0.105	0.014	0.77	0.081	0.004	0.075	0.008	0.74
DAG 38:3	0.389	0.061	0.428	0.040	0.53	0.396	0.042	0.276	0.045	0.08
DAG 38:4	5.825	0.689	6.794	0.640	0.25	6.159	0.595	4.474	0.658	0.07
DAG 38:5	0.504	0.071	0.518	0.057	0.83	0.427	0.033	0.343	0.048	0.26

Disease Models & Mechanisms • Supplementary information

DAG 38:6	0.533	0.066	0.532	0.041	0.99	0.561	0.054	0.379	0.036	0.02
DAG 38:7	0.055	0.002	0.054	0.005	0.91	0.059	0.006	0.059	0.005	0.94
DAG 38:8	0.093	0.005	0.101	0.005	0.47	0.103	0.010	0.087	0.006	0.17
DAG 40:4	0.199	0.023	0.245	0.033	0.28	0.201	0.029	0.177	0.019	0.62
DAG 40:5	0.035	0.002	0.037	0.002	0.74	0.039	0.002	0.035	0.004	0.42
DAG 40:6	0.303	0.040	0.295	0.024	0.82	0.241	0.023	0.202	0.025	0.31
DAG 40:7	0.099	0.011	0.106	0.008	0.57	0.095	0.008	0.078	0.007	0.21
DAG 40:8	0.034	0.002	0.038	0.005	0.32	0.032	0.004	0.030	0.003	0.74
DAG 42:0	0.001	0.000	0.001	0.000	0.97	0.001	0.000	0.001	0.000	0.58
DAG 42:1	0.018	0.003	0.018	0.002	0.84	0.014	0.002	0.016	0.003	0.62
DAG 42:2	0.014	0.003	0.015	0.001	0.59	0.012	0.002	0.017	0.003	0.18
DAG 42:6	0.705	0.045	0.781	0.053	0.42	0.713	0.098	0.687	0.071	0.80
DAG 42:7	0.411	0.011	0.441	0.014	0.22	0.401	0.025	0.396	0.009	0.85
DAG 44:0	0.009	0.001	0.008	0.001	0.59	0.008	0.001	0.008	0.001	0.88
DAG 44:1	0.001	0.000	0.002	0.000	0.65	0.002	0.000	0.001	0.000	**0.003**
DAG 44:2	0.023	0.002	0.026	0.003	0.47	0.025	0.003	0.022	0.002	0.38
DAG 44:8	0.276	0.022	0.280	0.010	0.88	0.275	0.020	0.279	0.020	0.89
Total DAG	31.465	2.772	32.436	2.296	0.77	29.147	2.723	27.764	2.670	0.71

Disease Models & Mechanisms • Supplementary information

Table S3. Diacylglycerol levels affected by XN in the hippocampus of FXR$^{Intestine-/-}$ mice.

	Female					Male				
FXR$^{Intestine-/-}$	HFD		XN		*P*-value	HFD		XN		*P*-value
Hippocampus	Mean	SEM	Mean	SEM		Mean	SEM	Mean	SEM	
DAG 30:0	0.007	0.001	0.007	0.001	0.79	0.007	0.001	0.005	0.001	0.48
DAG 30:1	0.002	0.000	0.003	0.001	0.20	0.002	0.000	0.003	0.000	0.72
DAG 30:2	0.007	0.001	0.008	0.001	0.33	0.009	0.001	0.008	0.002	0.58
DAG 32:0	0.117	0.010	0.128	0.013	0.59	0.102	0.008	0.116	0.013	0.52
DAG 32:1	0.441	0.035	0.421	0.053	0.76	0.372	0.053	0.358	0.028	0.86
DAG 32:2	0.007	0.001	0.007	0.001	0.99	0.008	0.001	0.005	0.001	0.09
DAG 32:3	0.006	0.001	0.007	0.001	0.54	0.008	0.001	0.006	0.001	0.37
DAG 34:0	0.528	0.059	0.534	0.067	0.95	0.533	0.084	0.442	0.040	0.40
DAG 34:1	2.053	0.170	1.816	0.242	0.48	1.914	0.310	1.681	0.126	0.54
DAG 34:2	1.226	0.078	1.176	0.143	0.82	1.285	0.258	1.145	0.080	0.57
DAG 34:3	3.974	0.413	4.062	0.517	0.90	3.773	0.544	3.262	0.179	0.51
DAG 36:1	0.684	0.074	0.703	0.175	0.92	0.537	0.101	0.555	0.067	0.94
DAG 36:2	2.375	0.252	2.204	0.289	0.66	2.296	0.315	2.042	0.175	0.56
DAG 36:3	5.742	0.498	5.574	0.646	0.86	5.438	0.779	4.928	0.243	0.64
DAG 36:4	1.891	0.141	2.001	0.218	0.74	2.007	0.365	1.935	0.122	0.85
DAG 36:5	0.032	0.005	0.029	0.004	0.63	0.044	0.009	0.033	0.001	0.18
DAG 36:6	0.050	0.008	0.046	0.004	0.68	0.043	0.008	0.046	0.007	0.71
DAG 38:0	0.065	0.008	0.067	0.012	0.84	0.061	0.004	0.061	0.011	0.98
DAG 38:1	0.059	0.010	0.061	0.014	0.88	0.054	0.008	0.051	0.005	0.87
DAG 38:2	0.068	0.008	0.072	0.018	0.82	0.066	0.007	0.067	0.008	0.96
DAG 38:3	0.312	0.022	0.300	0.081	0.87	0.287	0.094	0.245	0.032	0.62
DAG 38:4	4.920	0.343	4.575	1.032	0.73	4.485	1.336	4.286	0.567	0.86
DAG 38:5	0.397	0.040	0.379	0.062	0.82	0.362	0.086	0.338	0.043	0.78

DAG 38:6	0.474	0.045	0.465	0.047	0.91	0.461	0.096	0.416	0.067	0.62
DAG 38:7	0.056	0.005	0.068	0.007	0.21	0.058	0.010	0.056	0.011	0.82
DAG 38:8	0.082	0.007	0.101	0.008	0.13	0.094	0.008	0.095	0.005	0.94
DAG 40:4	0.148	0.016	0.203	0.052	0.28	0.197	0.062	0.143	0.020	0.35
DAG 40:5	0.033	0.002	0.035	0.004	0.74	0.030	0.004	0.030	0.003	0.97
DAG 40:6	0.229	0.032	0.203	0.029	0.53	0.200	0.028	0.221	0.035	0.66
DAG 40:7	0.080	0.012	0.081	0.009	0.95	0.065	0.008	0.083	0.010	0.27
DAG 40:8	0.031	0.005	0.039	0.004	0.17	0.027	0.003	0.036	0.005	0.17
DAG 42:0	0.002	0.001	0.002	0.000	0.55	0.001	0.000	0.001	0.000	0.47
DAG 42:1	0.012	0.002	0.011	0.003	0.70	0.015	0.002	0.014	0.003	0.88
DAG 42:2	0.012	0.003	0.011	0.003	0.77	0.009	0.001	0.012	0.002	0.55
DAG 42:6	0.685	0.057	0.664	0.094	0.85	0.633	0.104	0.606	0.032	0.83
DAG 42:7	0.409	0.024	0.410	0.020	0.95	0.383	0.009	0.386	0.019	0.93
DAG 44:0	0.006	0.001	0.007	0.001	0.95	0.008	0.001	0.008	0.001	0.76
DAG 44:1	0.001	0.000	0.002	0.000	0.21	0.002	0.000	0.001	0.000	0.05
DAG 44:2	0.020	0.003	0.024	0.002	0.43	0.022	0.002	0.018	0.001	0.35
DAG 44:8	0.279	0.026	0.313	0.025	0.28	0.259	0.024	0.261	0.015	0.96
Total DAG	27.523	1.757	26.818	3.454	0.86	26.159	3.986	24.007	1.373	0.64

Disease Models & Mechanisms • Supplementary information

Table S4. Ceramide and hexaceramide levels affected by XN in the hippocampus of wild-type mice.

	Fema le					Male				
Wild-type	HFD		XN		*P*-value	HFD		XN		*P*-value
Hippocampus	Mea n	SEM	Mean	SEM		Mean	SEM	Mean	SEM	
Cer(d32:1)	0.064	0.010	0.068	0.006	0.71	0.056	0.010	0.063	0.012	0.58
Cer(d34:1)	1.713	0.276	1.769	0.164	0.83	1.811	0.204	1.969	0.243	0.57
Cer(d36:1)	14.23 5	2.992	11.522	0.949	0.21	14.308	1.881	11.432	1.418	0.21
Cer(d36:2)	6.738	0.729	6.623	0.544	0.85	6.764	0.407	5.996	0.368	0.23
Cer(d38:1)	1.375	0.250	1.370	0.184	0.98	1.243	0.175	1.102	0.078	0.48
Cer(d38:2)	16.56 5	2.644	15.625	1.599	0.60	14.744	1.427	13.633	0.901	0.56
Cer(d40:1)	9.620	1.603	8.595	1.122	0.38	9.469	1.052	7.832	0.490	0.19
Cer(d40:2)	135.1 82	24.067	122.968	15.815	0.47	131.924	15.806	115.656	8.140	0.37
Cer(d42:1)	9.799	1.769	8.938	1.216	0.54	9.657	1.307	8.708	0.540	0.53
Cer(d42:2)	115.5 14	26.020	105.825	19.411	0.60	118.369	17.727	97.244	8.522	0.28
Total Ceramid	310.8 05	59.509	283.302	40.261	0.50	308.345	37.988	263.635	17.684	0.30
Hex2Cer(d34:1)	5.047	0.545	4.847	0.259	0.65	4.554	0.359	5.173	0.427	0.19
HexCer(d40:1)	25.34 1	3.720	22.452	2.471	0.32	26.234	3.006	21.487	1.351	0.13
HexCer(d42:1)	24.43 0	4.820	21.734	3.753	0.48	26.096	3.897	19.995	1.645	0.13
HexCer(d42:2)	226.7 35	55.211	205.331	41.523	0.57	229.673	35.735	185.410	15.010	0.28
Total HexCer	281.5 52	64.143	254.365	47.682	0.54	286.557	42.649	232.065	17.933	0.26

Disease Models & Mechanisms • Supplementary information

Table S5. Ceramide and hexaceramide levels affected by XN in the hippocampus of FXR$^{Liver-/-}$ mice.

	Female					Male				
FXR$^{Liver-/-}$	HFD		XN		*P*-value	HFD		XN		*P*-value
Hippocampus	Mean	SEM	Mean	SEM		Mean	SEM	Mean	SEM	
Cer(d32:1)	0.056	0.006	0.064	0.006	0.46	0.044	0.004	0.058	0.009	0.27
Cer(d34:1)	1.516	0.105	1.618	0.085	0.65	1.580	0.228	1.570	0.185	0.97
Cer(d36:1)	11.705	0.882	12.005	0.982	0.88	10.781	1.465	10.291	1.081	0.82
Cer(d36:2)	6.460	0.376	6.249	0.279	0.69	5.908	0.364	6.074	0.343	0.78
Cer(d38:1)	1.283	0.083	1.224	0.041	0.72	1.157	0.124	1.047	0.071	0.55
Cer(d38:2)	15.459	1.070	14.515	0.524	0.56	14.287	0.993	13.343	0.664	0.6
Cer(d40:1)	8.854	0.512	8.201	0.334	0.53	8.525	0.677	7.758	0.285	0.51
Cer(d40:2)	125.307	8.331	112.211	4.092	0.38	118.443	10.367	108.844	5.709	0.56
Cer(d42:1)	9.126	0.819	8.322	0.705	0.52	8.755	0.794	7.371	0.438	0.32
Cer(d42:2)	99.482	7.948	83.820	3.606	0.34	96.500	11.159	83.188	4.707	0.47
Total Ceramid	279.248	18.437	248.229	9.151	0.39	265.981	24.600	239.544	12.242	0.52
Hex2Cer(d34:1	4.496	0.185	4.856	0.155	0.36	4.470	0.263	4.712	0.298	0.58
HexCer(d40:1)	23.769	1.319	22.403	0.825	0.6	23.300	1.869	20.735	0.743	0.38
HexCer(d42:1)	22.502	1.929	18.599	0.726	0.25	21.358	2.586	17.967	0.682	0.37
HexCer(d42:2)	196.486	17.799	162.336	6.602	0.32	187.209	22.877	158.758	8.983	0.46
Total HexCer	247.254	20.910	208.194	8.099	0.33	236.337	27.330	202.172	10.451	0.45

Disease Models & Mechanisms • Supplementary information

Table S6. Ceramide and hexaceramide levels affected by XN in the hippocampus of FXR$^{Intestine-/-}$ mice.

	Female					Male				
FXR$^{Intestine-/-}$	HFD		XN		*P*-value	HFD		XN		*P*-value
Hippocampus	Mean	SEM	Mean	SEM		Mean	SEM	Mean	SEM	
Cer(d32:1)	0.061	0.013	0.054	0.011	0.61	0.057	0.011	0.069	0.015	0.44
Cer(d34:1)	1.408	0.135	1.642	0.207	0.39	1.695	0.123	1.961	0.219	0.4
Cer(d36:1)	12.518	2.026	11.544	0.891	0.67	13.236	1.466	10.437	1.400	0.28
Cer(d36:2)	6.336	0.211	5.921	0.164	0.52	7.089	0.686	6.191	0.491	0.22
Cer(d38:1)	1.145	0.127	1.169	0.052	0.9	1.450	0.157	1.252	0.143	0.38
Cer(d38:2)	14.605	1.117	14.417	0.385	0.92	16.798	1.584	14.968	1.120	0.4
Cer(d40:1)	8.621	0.907	8.135	0.182	0.7	9.990	1.396	8.853	0.809	0.42
Cer(d40:2)	117.757	11.287	116.390	2.425	0.94	139.524	14.039	126.062	10.590	0.51
Cer(d42:1)	8.830	0.785	8.152	0.445	0.65	10.425	1.666	9.402	1.062	0.55
Cer(d42:2)	97.178	13.296	92.978	3.662	0.83	117.143	15.638	104.695	9.578	0.58
Total Ceramid	268.459	27.460	260.403	7.130	0.85	317.407	33.538	283.888	23.587	0.5
Hex2Cer(d34:1	4.395	0.311	4.534	0.224	0.77	5.055	0.322	5.035	0.406	0.97
HexCer(d40:1)	24.163	2.473	21.797	0.488	0.45	26.968	3.636	24.987	1.735	0.57
HexCer(d42:1)	21.175	2.693	19.521	0.871	0.68	25.852	4.347	21.779	2.222	0.37
HexCer(d42:2)	184.081	24.731	177.294	8.528	0.87	224.151	29.494	201.201	19.912	0.62
Total HexCer	233.814	29.823	223.145	9.134	0.82	282.026	37.419	253.002	23.720	0.59

Table S7. Sphingolipid levels affected by XN in the hippocampus of wild-type mice.

	Female					Male				
Wild-type	HFD		XN		*P*-value	HFD		XN		*P*-value
Hippocampus	Mean	SEM	Mean	SEM		Mean	SEM	Mean	SEM	
SM(d30:1)	2.319	0.180	2.229	0.146	0.71	2.236	0.138	2.464	0.203	0.38
SM(d32:0)	2.268	0.121	2.286	0.105	0.91	2.051	0.127	2.073	0.162	0.90
SM(d32:1)	4.714	0.274	4.538	0.256	0.64	4.220	0.238	4.647	0.333	0.29
SM(d32:2)	2.460	0.217	2.564	0.203	0.69	1.883	0.149	2.127	0.170	0.38
SM(d33:1)	0.648	0.059	0.660	0.037	0.88	0.603	0.055	0.685	0.064	0.31
SM(d34:0)	5.747	0.369	5.580	0.195	0.70	5.591	0.349	5.925	0.431	0.46
SM(d34:1)	77.759	6.523	77.139	3.058	0.92	74.415	4.828	82.310	7.096	0.25
SM(d34:2)	4.825	0.524	4.982	0.310	0.82	5.505	0.903	5.287	0.498	0.76
SM(d36:0)	86.094	5.606	85.687	3.560	0.93	79.742	3.220	83.626	3.225	0.47
SM(d36:1)	1397.330	97.523	1430.080	78.994	0.74	1323.470	73.144	1472.230	88.669	0.15
SM(d36:2)	464.273	34.770	482.175	20.353	0.68	483.229	25.788	559.250	45.853	0.10
SM(d36:3)	3.994	0.579	3.769	0.219	0.54	3.496	0.202	3.790	0.219	0.45
SM(d38:1)	111.290	10.342	112.664	6.257	0.87	100.877	6.456	101.478	5.366	0.95
SM(d38:2)	3.081	0.199	3.151	0.144	0.81	3.023	0.197	3.171	0.276	0.63
SM(d39:1)	5.568	0.576	5.596	0.336	0.96	5.398	0.511	5.300	0.482	0.86
SM(d40:0)	4.031	0.410	3.906	0.299	0.72	3.947	0.313	3.596	0.260	0.35
SM(d40:1)	39.601	4.496	39.104	2.807	0.89	39.735	3.724	37.700	3.115	0.61
SM(d40:2)	20.800	1.615	21.506	1.377	0.71	18.679	1.232	19.076	1.813	0.84
SM(d41:1)	10.718	1.682	10.679	1.050	0.97	10.806	1.380	9.549	0.702	0.34
SM(d41:2)	9.920	1.066	10.651	1.184	0.58	8.499	0.812	8.491	1.028	0.99
SM(d42:1)	29.302	2.470	28.957	1.555	0.90	27.544	2.149	27.365	2.833	0.95
SM(d42:2)	279.064	28.818	289.710	18.877	0.73	261.149	25.666	271.421	33.235	0.76
SM(d43:1)	1.927	0.390	1.836	0.357	0.79	1.978	0.278	1.615	0.097	0.34

Disease Models & Mechanisms • Supplementary information

SM(d43:2)	3.522	0.385	3.818	0.337	0.51	3.505	0.355	3.161	0.351	0.47
SM(d44:2)	1.452	0.171	1.466	0.147	0.93	1.222	0.130	1.210	0.107	0.94
Total SM	2572.710	180.522	2634.730	98.693	0.73	2472.800	146.975	2717.550	193.041	0.20
sphinganine d17:0	0.565	0.071	0.394	0.049	0.12	0.368	0.055	0.664	0.106	**0.01**
CH3sphinganine d18:0	0.032	0.004	0.028	0.002	0.6	0.030	0.006	0.035	0.003	0.55
Total Sphinganine	0.597	0.073	0.422	0.050	0.13	0.398	0.056	0.699	0.108	0.02
sphingosine d18:1	1.141	0.150	0.964	0.045	0.15	0.993	0.099	1.013	0.130	0.88
sphingosine d20:1	0.186	0.029	0.159	0.013	0.27	0.154	0.018	0.174	0.029	0.46
Total Sphingosine	1.327	0.179	1.122	0.053	0.16	1.147	0.114	1.187	0.158	0.80

SM: Sphingomyelin.

Disease Models & Mechanisms • Supplementary information

Table S8. Sphingolipid levels affected by XN in the hippocampus of FXR$^{Liver–/–}$ mice.

	Female					Male				
FXR$^{Liver–/–}$	HFD		XN		*P*-value	HFD		XN		*P*-value
Hippocampus	Mean	SEM	Mean	SEM		Mean	SEM	Mean	SEM	
SM(d30:1)	2.104	0.130	2.189	0.178	0.70	1.934	0.154	1.986	0.140	0.83
SM(d32:0)	2.127	0.087	2.095	0.074	0.83	1.924	0.126	1.986	0.078	0.72
SM(d32:1)	4.356	0.229	4.538	0.263	0.59	4.267	0.205	4.098	0.211	0.66
SM(d32:2)	2.492	0.113	2.204	0.220	0.22	2.320	0.112	2.292	0.134	0.92
SM(d33:1)	0.637	0.035	0.726	0.036	0.20	0.684	0.084	0.566	0.051	0.12
SM(d34:0)	5.694	0.184	5.876	0.287	0.63	5.660	0.313	5.493	0.307	0.69
SM(d34:1)	73.962	2.398	80.268	3.167	0.27	76.562	4.801	73.192	4.688	0.60
SM(d34:2)	4.709	0.179	4.895	0.304	0.76	5.621	0.589	4.880	0.402	0.27
SM(d36:0)	84.620	2.073	85.881	3.546	0.78	79.114	2.468	82.881	3.453	0.45
SM(d36:1)	1322.210	60.151	1359.450	29.214	0.67	1314.840	70.460	1349.150	69.146	0.42
SM(d36:2)	460.260	19.295	501.904	27.574	0.29	493.001	34.009	499.701	25.959	0.88
SM(d36:3)	3.337	0.230	3.548	0.139	0.51	3.310	0.202	3.222	0.144	0.81
SM(d38:1)	110.467	3.937	110.496	3.289	0.99	106.130	6.341	100.052	4.924	0.46
SM(d38:2)	3.326	0.155	3.002	0.206	0.21	3.265	0.113	3.227	0.160	0.90
SM(d39:1)	5.461	0.238	5.707	0.280	0.61	5.431	0.372	5.104	0.349	0.54
SM(d40:0)	3.606	0.100	3.795	0.230	0.54	3.765	0.233	3.582	0.148	0.60
SM(d40:1)	35.844	1.006	36.957	1.595	0.74	38.240	2.967	34.954	2.000	0.38
SM(d40:2)	19.643	0.843	21.472	0.943	0.28	20.581	1.473	19.412	1.330	0.54
SM(d41:1)	9.820	0.397	10.021	0.511	0.85	9.984	0.750	9.132	0.586	0.49
SM(d41:2)	10.221	0.950	11.119	0.919	0.45	9.286	0.870	8.678	0.672	0.64
SM(d42:1)	27.871	1.705	30.524	1.917	0.30	26.590	1.480	26.320	1.577	0.92
SM(d42:2)	266.511	14.191	284.529	16.614	0.51	254.357	19.151	249.049	20.027	0.86
SM(d43:1)	1.940	0.193	1.662	0.168	0.38	1.973	0.307	1.565	0.137	0.25
SM(d43:2)	3.551	0.267	4.013	0.310	0.25	3.399	0.296	3.104	0.288	0.51

Disease Models & Mechanisms • Supplementary information

SM(d44:2)	1.347	0.105	1.338	0.076	0.95	1.233	0.100	1.160	0.110	0.65
Total SM	2466.120	78.332	2578.210	76.465	0.48	2473.470	135.262	2494.790	128.388	0.90
sphinganine d17:0	0.388	0.059	0.676	0.073	**0.004**	0.357	0.064	0.529	0.063	0.12
CH3sphinganine d18:0	0.034	0.006	0.041	0.004	0.29	0.028	0.004	0.037	0.007	0.23
Total Sphinganine	0.421	0.062	0.717	0.076	**0.005**	0.385	0.068	0.567	0.067	0.11
sphingosine d18:1	0.955	0.044	1.059	0.040	0.34	0.911	0.094	1.061	0.063	0.22
sphingosine d20:1	0.166	0.007	0.187	0.012	0.34	0.152	0.021	0.158	0.007	0.82
Total Sphingosine	1.121	0.045	1.246	0.048	0.33	1.063	0.114	1.219	0.067	0.28

SM: Sphingomyelin.

Table S9. Sphingolipid levels affected by XN in the hippocampus of $FXR^{Intestine-/-}$ mice.

	Female					Male				
$FXR^{Intestine-/-}$	HFD		XN		*P*-value	HFD		XN		*P*-value
Hippocampus	Mean	SEM	Mean	SEM		Mean	SEM	Mean	SEM	
SM(d30:1)	1.903	0.097	2.258	0.137	0.18	2.888	0.238	2.443	0.366	0.13
SM(d32:0)	1.957	0.087	1.867	0.085	0.62	2.287	0.270	2.139	0.215	0.47
SM(d32:1)	4.165	0.151	4.526	0.184	0.37	4.954	0.442	4.675	0.520	0.54
SM(d32:2)	2.051	0.121	1.940	0.125	0.69	2.600	0.322	2.140	0.349	0.15
SM(d33:1)	0.635	0.059	0.631	0.072	0.96	0.756	0.041	0.603	0.053	0.10
SM(d34:0)	5.525	0.295	5.509	0.233	0.97	5.914	0.432	5.600	0.345	0.54
SM(d34:1)	72.005	4.753	74.174	3.987	0.75	82.698	4.951	75.749	5.374	0.37
SM(d34:2)	5.247	0.574	5.172	0.397	0.92	6.010	0.695	5.197	0.509	0.32
SM(d36:0)	80.245	2.186	82.893	3.103	0.62	89.416	6.597	83.611	3.628	0.34
SM(d36:1)	1281.750	53.680	1355.280	50.573	0.48	1465.540	86.703	1320.240	76.481	0.22
SM(d36:2)	471.740	28.098	481.837	30.359	0.83	495.466	47.987	463.654	45.734	0.55
SM(d36:3)	3.298	0.162	3.588	0.159	0.45	3.830	0.258	3.457	0.295	0.40
SM(d38:1)	94.637	5.427	97.177	3.808	0.77	111.497	7.062	102.911	7.342	0.39
SM(d38:2)	3.033	0.248	2.462	0.137	0.07	3.177	0.323	3.081	0.368	0.78
SM(d39:1)	5.004	0.316	4.979	0.346	0.96	5.981	0.412	5.271	0.416	0.28
SM(d40:0)	3.671	0.189	3.335	0.185	0.36	4.241	0.357	3.812	0.218	0.31
SM(d40:1)	34.889	2.107	34.199	2.403	0.86	42.354	3.011	38.399	2.715	0.39
SM(d40:2)	18.504	1.358	18.463	1.380	0.98	20.776	1.619	20.748	1.764	0.99
SM(d41:1)	8.816	0.599	9.326	0.557	0.70	11.037	0.923	10.320	0.869	0.63
SM(d41:2)	8.743	0.811	8.819	0.930	0.96	9.385	0.834	9.443	0.961	0.97
SM(d42:1)	26.984	1.774	25.827	2.351	0.70	27.951	2.359	28.382	2.547	0.90
SM(d42:2)	245.521	18.642	250.117	24.192	0.89	269.432	27.303	273.816	25.120	0.91
SM(d43:1)	1.739	0.189	1.709	0.245	0.94	1.914	0.258	1.755	0.254	0.71

Disease Models & Mechanisms • Supplementary information

SM(d43:2)	3.053	0.230	3.228	0.337	0.71	3.484	0.270	3.446	0.364	0.94
SM(d44:2)	1.143	0.064	1.157	0.136	0.94	1.289	0.121	1.235	0.121	0.79
Total SM	2386.260	103.851	2480.480	93.878	0.62	2674.870	160.714	2472.120	156.492	0.35
sphinganine d17:0	0.538	0.102	0.612	0.089	0.53	0.621	0.136	0.486	0.105	0.31
CH3sphinganine d18:0	0.037	0.005	0.034	0.003	0.72	0.046	0.006	0.037	0.013	0.36
Total Sphinganine	0.576	0.107	0.647	0.091	0.56	0.667	0.139	0.524	0.117	0.30
sphingosine d18:1	0.886	0.100	0.950	0.077	0.63	1.074	0.105	0.955	0.092	0.42
sphingosine d20:1	0.159	0.018	0.160	0.016	0.99	0.174	0.017	0.166	0.023	0.79
Total Sphingosine	1.046	0.113	1.110	0.086	0.68	1.249	0.120	1.121	0.113	0.46

SM: Sphingomyelin.
